# Bacteriology and Changes in Antibiotic Susceptibility in Adults with Community-Acquired Perforated Appendicitis

**DOI:** 10.1371/journal.pone.0111144

**Published:** 2014-10-24

**Authors:** Hong Gil Jeon, Hyeong Uk Ju, Gyu Yeol Kim, Joseph Jeong, Min-Ho Kim, Jae-Bum Jun

**Affiliations:** 1 Department of Internal Medicine, Ulsan University Hospital, University of Ulsan College of Medicine, Ulsan, Republic of Korea; 2 Department of Surgery, Ulsan University Hospital, University of Ulsan College of Medicine, Ulsan, Republic of Korea; 3 Department of Laboratory Medicine, Ulsan University Hospital, University of Ulsan College of Medicine, Ulsan, Republic of Korea; 4 Biomedical Research Center, Ulsan University Hospital, University of Ulsan College of Medicine, Ulsan, Republic of Korea; University of Ottawa, Canada

## Abstract

This study evaluated bacterial etiology and antibiotic susceptibility in patients diagnosed with community-acquired perforated appendicitis over a 12-year-period. We retrospectively reviewed records of adult patients diagnosed with perforated appendicitis at an 800-bed teaching hospital between January 2000 and December 2011. In total, 415 culture-positive perforated appendicitis cases were analyzed. *Escherichia coli* was the most common pathogen (277/415, 66.7%), followed by *Streptococcus* species (61/415, 14.7%). The susceptibility of *E. coli* to ampicillin, piperacillin/tazobactam, ceftriaxone, cefepime, amikacin, gentamicin, and imipenem was 35.1%, 97.1%, 97.0%, 98.2%, 98.9%, 81.8%, and 100%, respectively. The overall susceptibility of *E. coli* to quinolones (ciprofloxacin or levofloxacin) was 78.7%. During the study period, univariate logistic regression analysis showed a significant decrease in *E. coli* susceptibility to quinolones (OR = 0.91, 95% CI 0.84–0.99, *P* = 0.040). We therefore do not recommend quinolones as empirical therapy for community-acquired perforated appendicitis.

## Introduction

Acute appendicitis is one of the most common abdominal surgical emergencies; it is also typically a community-acquired infection. Despite the generally favorable outcome, complicated appendicitis, such as perforated appendicitis, is associated with increased morbidity compared with simple acute appendicitis [1], [2]. Because *Escherichia coli* and *Bacteroides fragilis* are most commonly associated with appendicitis, antibiotic therapies are generally selected to target these bacteria [3], [4].

For adult patients with community-acquired complicated intra-abdominal infections of mild-to moderate severity, the use of ticarcillin-clavulanate, cefoxitin, ertapenem, moxifloxacin, or tigecycline as single-agent therapy or combinations of metronidazole with cefazolin, cefuroxime, ceftriaxone, cefotaxime, levofloxacin, or ciprofloxacin are recommended by Infectious Diseases Society of America (IDSA) guidelines [5]. However, with increased *E. coli* resistance to quinolones, investigation of local microbiologic findings had been proposed when selecting empirical therapies [5].

Because previous literature has reported a proportionally greater ratio of extended-spectrum β-lactamase (ESBL) and quinolone-resistant *E. coli* among bacteria responsible for community-acquired abdominal infections in Asia compared to other regions, careful selection of empirical antibiotics is particularly important in Asia [6]–[8]. We therefore conducted a study of the local microbiological profile and changes in antibiotic resistance in community-acquired perforated appendicitis over the past 12 years. These results may help us to inform selection of empirical antibiotic treatments for community-acquired complicated appendicitis.

## Materials and Methods

### Patient selection and data collection

We retrospectively reviewed the records of adult patients (age ≥18 years) who were diagnosed to have perforated appendicitis at Ulsan University Hospital, an 800-bed teaching hospital, between January 2000 to December 2011. Hospital charts and follow-up records were reviewed.

### Definitions

Perforated appendicitis was defined as either gross or microscopic evidence of appendiceal perforation. The appendix was not considered to be ruptured by the mere presence of suppurative peritoneal fluid or gangrenous appendicitis without microscopic evidence of perforation.

Community-acquired appendicitis was defined as appendicitis that occurred within 48 hours of hospital admission. Patients were excluded from the study if they had at least 1 of the following health care risk factors: 1) presence of an invasive device at time of admission, 2) history of MRSA infection or colonization, 3) history of surgery, hospitalization, dialysis, or residence in a long-term care facility in the 12 months preceding the culture date [5].

Systemic inflammatory response syndrome (SIRS), sepsis, severe sepsis, and septic shock were defined as described elsewhere [9].

### Specimen culture, species identification, and susceptibility testing

Specimens were obtained by swabbing the suppurative peritoneal fluid or periappendiceal abscess. In some cases, specimens were obtained by swabbing the lumen of appendix or by retrieving the suppurative peritoneal fluid via syringe aspiration. The swab specimens were transported to the laboratory in a transport medium (Amies transport medium without Charcoal; Asan Pharmaceuticals Co., Ltd., Hwasung, Korea). The specimens were either dispatched to the microbiology laboratory directly or stored in the operating room until the next day if collected after the working hours. The specimens were inoculated on blood agar, chocolate agar, and MacConkey agar plates. Samples were not inoculated into anaerobic culture. An automated VITEK 2 system (bioMerieux, Inc. Durham, NC, USA) was used to identify pathogens and perform ESBL susceptibility testing. The Vitek 2 ESBL test has 6 wells containing cefepime at 1 µg/mL, cefotaxime at 0.5 µg/mL, and ceftazidime at 0.5 µg/mL alone and in combination with clavulanic acid (10 µg/mL, 4 µg/mL, and 4 µg/mL, respectively); growth rate in each well is quantitatively assessed with an optical scanner. The proportional growth reduction (over 50%) in wells containing cephalosporin plus clavulanic acid compared with those containing cephalosporin alone was considered evidence of ESBL production. Susceptibility testing results were interpreted according to the National Committee for Clinical Laboratory Standards (CLSI) guidelines published in 2009 [10]. However, cephalosporin susceptibility results of ESBL-positive strains were interpreted on the basis of the strains’ respective minimal inhibitory concentration (MIC) breakpoints.

### Ethics statement

This retrospective study was approved by the Institutional Review Board (IRB) committee of Ulsan University Hospital. Written consent given by the patients was waived by the approving IRB.

### Statistical analysis

Statistical analyses were performed by using IBM SPSS Statistics for Windows, version 21 (IBM Corp., Armonk, NY, USA). The Chi-squared test was used to compare frequencies. A univariate logistic regression model was used to calculate odds ratios (OR), 95% confidence intervals, and p-values. The significance level was set at 0.05.

## Results

### Study population and clinical characteristics

Of 3,379 patients, 567 (16.7%) who received appendectomies during the study period were diagnosed with perforated appendicitis. Of these, we discarded 4 cases of health care-associated infection, 6 without confirmatory cultures, and 142 culture-negative cases; in total, we analyzed 415 culture-positive perforated appendicitis cases. The average length of hospitalization was 9.1±5.1 days. Patient ages ranged between 20 years and 94 years (mean 48.6±17.0 years), with 51.1% (212/415) men (Table 1). A majority of patients (404, 97.3%) underwent open appendectomy via a McBurney incision; laparotomy with a low midline incision was performed in 9 patients (2.2%) and laparoscopic appendectomy was performed in 2 patients (0.5%). The most common underlying disease was hypertension, reported in 56 patients (13.5%). Severe sepsis or septic shock was observed in 70 patients (16.8%), while 1 patient (0.2%) died of sepsis after mechanical ileus. Post-operative complications included wound infection in 18 patients (4.3%), abdominal abscesses or peritonitis in 7 patients (1.6%), and mechanical ileus in 6 patients (1.4%). A combination therapy comprising cephalosporin and metronidazole was the most frequent empirical antibiotic treatment.

**Table 1:** Baseline characteristics of patients with perforated appendicitis.

Characteristics	Number (%)
Number of culture positive patients	415
Hospital day (Mean ± SD)	9.1±5.1
Age (Mean ± SD)	48.6±17.0
Sex	
male	212 (51.1)
female	203 (48.9)
Operation method	
laparoscopic	2 (0.5)
McBurney	404 (97.3)
laparotomy	9 (2.2)
Underlying disease	
hypertension	56 (13.5)
diabetes mellitus	26 (6.3)
hepatitis B virus	16 (3.9)
solid cancer	12 (2.9)
Initial manifestation	
infection without SIRS	73 (17.6)
sepsis	272 (65.7)
severe sepsis	67 (16.1)
septic shock	3 (0.7)
In-hospital mortality	
alive	414 (99.8)
death	1 (0.2)
Infectious complication	
wound infection	18 (4.3)
intra-abdominal abscess or peritonitis	7 (1.6)
mechanical ileus	6 (1.4)
Antibiotics	
1st (or 2nd) generation cephalosporin + metronidazole	215 (51.8)
3rd generationcephalosporin + metronidazole	193 (46.5)
ciprofloxacin + metronidazole	4 (1.0)
piperacillin/tazobactam	3 (0.7)

SIRS = systemic inflammatory response syndrome.

### Microbiological features

The most commonly isolated bacteria was *E. coli* (277 isolates, 66.7%), followed by *Streptococcus* spp. (61, 14.7%), *Enterococcus* spp. (32, 7.7%), *Klebsiella* spp. (25, 6.0%), and *Pseudomonas aeruginosa* (24, 5.8%) (Table 2). More than 2 organisms were isolated in 75 cases (18.0%).

**Table 2:** Distribution of bacterial species.

	Species	Number (%)^f^
Gram negative organism	*Escherichia coli*	277 (66.7)
	*Klebsiella* species^a^	25 (6.0)
	*Pseudomonas aeruginosa*	24 (5.8)
	Other gramnegative organism^b^	45 (10.8)
Gram positive organism	*Streptococcus* species^c^	61 (14.7)
	*Enterococcus* species^d^	32 (7.7)
	*Staphylococcus aureus*	6 (1.4)
	Other gram positive organism^e^	23 (5.5)

a Includes: *K. pneumoniae, K. oxytoca*.

b Includes: *Achromobacter xylosoxidans, Acinetobacter lwoffii, Aeromonas hydrophila, Comamonas testosteroni, Hafnia alvei, Proteus mirabilis, Raoultella planticola, Serratia* species, *Enterobacter cloacae*.

c Includes: *S. alactolyticus, S. anginosus, S. cristatus, S. constellatus, S. gordonii, S. intermedius, S. mitis, S. salivarius, S. sanguinis*, Viridans Streptococci.

d Includes: *E. avium, E. faecalis, E. faecium, E. gallinarum, E. hirae, E. raffinosus*.

e Includes: *Gemella morbillorum, Lactococcus garvieae, Leuconostoc mesenteroides, Pediococcus pentosaceus*.

f Polymicrobial infection: 75 cases (18.0%).

### Antibiotic susceptibilities of isolated organisms

Data on antibiotic susceptibilities of isolated organisms showed that *E. coli* had 78.7% susceptibility to quinolones (ciprofloxacin or levofloxacin). Susceptibilities to ampicillin, aztreonam, ampicillin/sulbactam, amoxicillin/clavulanic acid, piperacillin/tazobactam, cefazolin, cefoxitin, ceftriaxone, cefepime, trimethoprim/sulfamethoxazole, amikacin, gentamicin, tobramycin, and imipenem were 35.1%, 95.2%, 41.4%, 83.5%, 97.1%, 89.8%, 97.7%, 97.0%, 98.2%, 65.6%, 98.9%, 81.8%, 83.4%, and 100%, respectively (Table 3). ESBL-producing strains accounted for 3.9% of *E*. *coli* species. *Streptococcus* species showed 68.9% susceptibility to penicillin, and 100% susceptibility to ceftriaxone. *Enterococcus* species were 71.9% susceptible to penicillin. The susceptibilities of *P. aeruginosa* to piperacillin/tazobactam, cefepime, quinolones, amikacin, and imipenem were 95.2%, 100%, 87.5%, 100%, and 95.8%, respectively.

**Table 3:** Antibiotic susceptibilities of isolated organisms that caused perforated appendicitis.

	*E. coli* (total)	*E. coli* (non-ESBL)	*E. coli* (ESBL)	*Streptococcus*species	*Enterococcus* species	*P. aeruginosa*
*Antibiotic*	(n = 277)	(n = 266)	(n = 11)	(n = 61)	(n = 32)	(n = 24)
Penicillin				42/61(68.9)	23/32(71.9)	
Ampicillin	97/276 (35.1)	97/265 (36.6)	0/11(0)		24/27(88.8)	
Aztreonam	220/231 (95.2)	217/220 (98.6)	3/11(27.2)			
Ampicillin/sulbactam	84/203 (41.4)	84/200 (42.0)	0/3 (0)			
Amoxicillin/clavulanic acid	61/73 (83.5)	55/65 (84.6)	6/8(75.0)			
Piperacillin/tazobactam	240/247 (97.1)	229/236 (97.0)	11/11 (100)			20/21 (95.2)
Cefazolin	248/276 (89.8)	248/265 (93.5)	0/11(0)			
Cefoxitin	264/270 (97.7)	254/259 (98.0)	10/11 (90.9)			
Ceftriaxone	267/275 (97.0)	262/264 (99.2)	5/11(45.4)	39/39(100)		2/24 (8.3)
Cefepime	227/231 (98.2)	220/220 (100)	7/11(63.6)			22/22 (100)
Quinolone	218/277 (78.7)	215/266 (80.8)	3/11(27.2)		25/28(89.2)	21/24 (87.5)
Trimethoprim/sulfamethoxazole	181/276 (65.6)	177/265 (66.7)	4/11(36.3)	34/47(72.3)	11/17(64.7)	1/24 (4.1)
Amikacin	274/277 (98.9)	264/266 (99.2)	10/11 (90.9)			24/24 (100)
Gentamicin	226/276 (81.8)	221/265 (83.4)	5/11(45.4)			24/24 (100)
Tobramycin	231/277 (83.4)	227/266 (85.3)	4/11(36.3)			24/24 (100)
Vancomycin				60/61(98.3)	30/32(93.7)	
Imipenem	276/276 (100)	265/265 (100)	11/11 (100)	11/11(100)	23/26(88.5)	23/24 (95.8)

### Comparisons of bacterial species and *E. coli* isolate antibiotic susceptibilities by clinical severity

We compared the bacterial species and antibiotic susceptibilities of *E. coli* isolates according to the clinically indicated severity (Table 4). The cases were redistributed into two major groups: “sepsis” and “severe sepsis.” Infected patients without SIRS and the patients with sepsis were grouped together in the “sepsis” group, whereas the patients with severe sepsis and septic shock were grouped together in the “severe sepsis” group. A total of 345 patients (83.1%) were included in the sepsis group and 70 (16.9%) were included in the severe sepsis group. *E. coli* isolates were found more frequently in the severe sepsis group (74.3%) than in the sepsis group (65.2%), but the difference was not statistically significant. The isolation rates of the other species were also not significantly different between groups. There were no statistically significant differences in *E. coli* susceptibility to all antibiotics between groups.

**Table 4:** Comparisons of bacterial species and antibiotic susceptibilities of *E. coli* between the sepsis group and the severe sepsis group.

	Sepsis^a^ (%)	Severe sepsis^b^ (%)	*P*-value
Species			
* E. coli*	225/345 (65.2)	52/70 (74.3)	0.142
* P. aeruginosa*	20/345 (5.8)	4/70 (5.7)	0.978
* Streptococcus* species	52/345 (15.1)	9/70 (12.9)	0.633
* Enterococcus* species	25/345 (7.3)	7/70 (10.0)	0.431
Antibiotics susceptibilities of *E. coli*			
* *Piperacillin/tazobactam	197/202 (97.5)	43/45 (95.6)	0.472
* *Cefoxitin	214/220 (97.3)	50/50 (100)	0.238
* *Ceftriaxone	215/223 (96.4)	52/52 (100)	0.166
* *Cefepime	184/187 (98.4)	43/44 (97.7)	0.760
* *Ciprofloxacin or levofloxacin	176/225 (78.2)	42/52 (80.8)	0.686

a Infection without SIRS (systemic inflammatory response syndrome) & sepsis.

b Severe sepsis & septic shock.

### Changes in *E. coli* antimicrobial susceptibility according to the year

**Figure 1:**
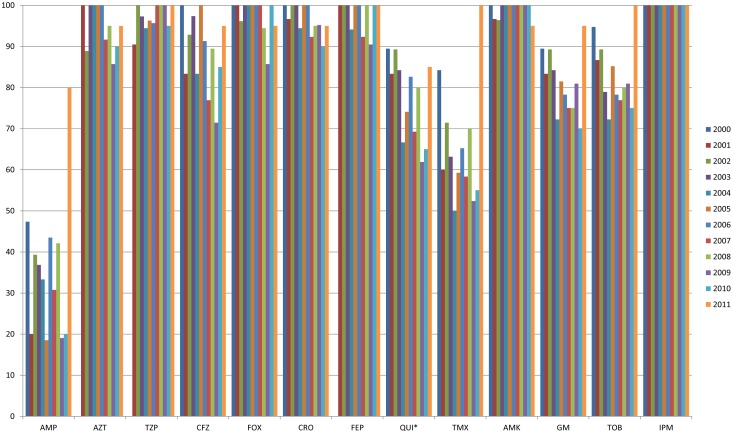
Change of antimicrobial susceptibility among *E. coli* during the 12-year-period. AMP, ampicillin; AZT, aztreonam; TZP, piperacillin/tazobactam; CFZ, cefazolin; FOX, cefoxitin; CRO, ceftriaxone; FEP, cefepime; QUI, quinolone; TMX, trimethoprim/sulfamethoxazole; AMK, amikacin; GM, gentamicin; TOB, tobramycin; IPM, imipenem. * During the study period, there was a significant decrease in antimicrobial susceptibility on univariate logistic regression analysis (*P* = 0.040).

Yearly changes in *E. coli* antimicrobial susceptibility during the study period were examined (Fig. 1). Univariate logistic regression analysis showed that *E. coli* susceptibility to quinolones significantly decreased, with annual susceptibility rates of 89.4%, 83.3%, 89.2%, 84.2%, 66.6%, 74.0%, 82.6%, 69.2%, 80.0%, 61.9%, 65.0%, and 85.0%, during the period of 2000 to 2011 (OR = 0.91, 95% CI 0.84–0.99, *P* = 0.040). In particular, *E. coli* susceptibility to cefoxitin (*P* = 0.052) and ceftriaxone (*P* = 0.054) decreased during the study period, but the change was not statistically significant. Nor were any statistically significant changes observed in *E. coli* susceptibility to other antibiotics such as ampicillin (*P* = 0.235), aztreonam (*P* = 0.168), piperacillin/tazobactam (*P* = 0.645), cefazolin (*P* = 0.126), cefepime (*P* = 0.393), trimethoprim/sulfamethoxazole (*P* = 0.732), amikacin (*P* = 0.835), gentamicin (*P* = 0.389), and tobramycin (*P* = 0.645).

## Discussion

This study evaluated microbiological profiles and antibiotic susceptibilities of pathogens isolated from cases of perforated appendicitis. The flora detected in complicated intra-abdominal infection differs between community-acquired and nosocomial infections. We considered appendicitis suitable for studying community-acquired bacterial infections since this illness is largely community-acquired. In fact, only 4 patients discarded from analysis owing to health care-associated infections. *P. aeruginosa* isolates in this study showed overall high levels of antibiotic susceptibility with no multidrug-resistant strains, supporting the idea that appendicitis is more commonly a community-acquired rather than nosocomial infection [11].

*E. coli* was the most common pathogen identified in this study (66.7% of all isolates), similar to findings in previous appendicitis literature [3], [12]. Similarly, *Streptococcus* and *Enterococcus* species were the most frequently isolated gram-positive organisms [12], [13]. The ratio of ESBL-producing *E. coli* was 3.9%, within previously reported ranges of 3.5–15.4% [8], [14]. The isolation rate of *E. coli* was greater in the severe sepsis group, although this difference was not statistically significant. Some studies have reported that *P. aeruginosa* is a commonly isolated strain in appendicitis, with an isolation rate of 19–32%; however, this was not the case in the current study [12], [15].

Although *E. coli* showed a high susceptibility rate of 97% to second- and third-generation cephalosporins that are most commonly used for empirical antibiotic treatment, the susceptibility decreased during the study period, albeit without statistical significance (*P* = 0.052 and *P* = 0.054, respectively). The susceptibility to quinolones was 78.7%, with a statistically significant (*P* = 0.040) decrease during the study period. Previous studies by Bochicchio et al (2006) and Rob et al (2013) reported that the susceptibility rate of *E. coli*, isolated from appendicitis samples, to quinolones was 71.4–85.6% [6], [8]. The *E. coli* susceptibility to quinolones and cephalosporins reported by Rob et al (2013) was lower than that reported by Bochicchio et al (2006). This may be attributable to *E. coli*’s increased resistance to the antibiotics or the lowered MIC breakpoint for cephalosporins set by the CLSI guidelines. For most antibiotics, *E. coli* susceptibility rates observed in this study were similar to those reported by Bochicchio et al (2006), with the susceptibility rate to quinolones being slightly lower. Both previous studies found high susceptibilities to carbapenem, amikacin, and piperacillin/tazobactam; in this study, ESBL-producing organisms were particularly sensitive to piperacillin/tazobactam (12/12, 100%). The susceptibility of *Streptococcus* species to penicillin was 68.9%, and all strains were susceptible to ceftriaxone. *P. aeruginosa* isolated in this study was highly susceptible to amikacin, cefepime, piperacillin/tazobactam, and carbapenem, but was slightly less susceptible to quinolones (87.5%).

All patients undergoing operation for appendicitis should receive antimicrobial therapy [16]. Appropriate antimicrobial therapy includes agents effective against facultative and aerobic gram-negative organisms and anaerobic organisms. There are data that inadequate empiric antibiotic therapy results in increased morbidity or treatment failure in complicated appendicitis [17], [18]. If resistance to a given antibiotic is present in 10%–20% or more of isolates of a common intra-abdominal pathogen in the community, use of that agent should be avoided [5]. A report in Taiwan proposed that a quinolone be used to treat community-acquired complicated intra-abdominal infections, as *E. coli* was found to be 82–85% susceptible to ciprofloxacin and levofloxacin [19]. In this study, however, the resistance rate of *E. coli* to quinolones is >20%; therefore, its use as an empirical antibiotic is not advisable in Korea. Second- and third-generation cephalosporins appeared to be an appropriate treatment for this application according to our results. Although third-generation cephalosporins might be a better treatment choice because that *Streptococcus* species showed 100% susceptibility to ceftriaxone, further studies are needed to thoroughly trace variations in susceptibility, given that the decrease in *E. coli* susceptibility, observed during the study period, was not statistically significant. Piperacillin/tazobactam and carbapenem might be considered to treat *P. aeruginosa* or ESBL-producing organisms in patients with signs of severe sepsis such as organ dysfunction. However, these species were not frequently isolated in all patient groups including the severe sepsis group of the current study, and spectrum of these antibiotics may be too broad. *E. coli* also showed high susceptibility to amikacin, but concerns remain regarding use of aminoglycoside antibiotics owing to their nephrotoxicity and ototoxicity. Considering the high resistance of *E. coli* to ampicillin and ampicillin/sulbactam–and the questionable significance of enterococci as pathogens in complicated intra-abdominal infections–these antibiotics are not recommended for treating perforated appendicitis.

On the basis of evidence that culture testing of intraoperative specimens does not affect the prognosis of patients with perforated appendicitis, many institutions may not perform routine culture testing [20], [21]. However, considering the current reality of increasing antibiotic resistance, routine culture testing might be useful to identify changes in susceptibility and to select appropriate antibiotics [5]. Anaerobic cultures are not necessary for patients with community-acquired intra-abdominal infection if empiric antimicrobial therapy active against common anaerobic pathogens is provide [5]. Although anaerobic bacteria culturing was not performed in this study, previous reports on anaerobic culture showed that *Bacteroides fragilis*, along with *E. coli*, was the most commonly isolated pathogen in appendicitis [14], [20]. In past studies of appendicitis that conducted anaerobic susceptibility testing, *B. fragilis* was found to be more than 95% susceptible to metronidazole [4], [14], [22]. Anaerobic bacteria culturing could be considered for future studies if an increase in anaerobic bacterial resistance to metronidazole is observed.

The retrospective nature of the present study might have resulted in intrinsic bias and the data may not represent the entire population because data was collected from a single institution. However, considering that the quinolone resistance rate we observed was similar to that reported in previous studies conducted in Korea [23], [24]–which involved community-acquired *E. coli* bacteremia originated from various infections including intra-abdominal infection–we speculated that quinolone resistance rate among *E. coli* causing intra-abdominal infection in Korea should be similar to the one determined in this study.

In conclusion, *E. coli* was the most commonly identified pathogen in patients with perforated appendicitis. The quinolone resistance rate was >20% in *E. coli* isolated from community-acquired perforated appendicitis. The isolates were decreasingly susceptible to quinolones during the study period. We advise against the use of quinolones as a first line antibiotic therapy in community-acquired perforated appendicitis in Korea.
